# Double-Stranded DNA in Exosomes of Malignant Pleural Effusions as a Novel DNA Source for EGFR Mutation Detection in Lung Adenocarcinoma

**DOI:** 10.3389/fonc.2019.00931

**Published:** 2019-09-25

**Authors:** Xueling Qu, Qiuwen Li, Jingwen Yang, Huixia Zhao, Feifei Wang, Fengyun Zhang, Shufang Zhang, He Zhang, Ruliang Wang, Qian Wang, Qi Wang, Guanghui Li, Xiumei Peng, Xuan Zhou, Yixin Hao, Jianhua Zhu, Wenhua Xiao

**Affiliations:** ^1^Department of Oncology, The Fourth Medical Center of PLA General Hospital, Beijing, China; ^2^Department of Oncology, People's Hospital of Beijing Daxing District, Capital Medical University, Beijing, China; ^3^Department of Oncology, The First Hospital of Hebei Medical University, Shijiazhuang, China; ^4^Department of Internal Medicine, Sino-Singapore Eco-City Hospital of Tianjin Medical University, Tianjin, China

**Keywords:** exosome, double-stranded DNA, EGFR mutation, malignant pleural effusions, lung adenocarcinoma

## Abstract

**Background:** Exosomes are cell-derived vesicles and bear a specific set of nucleic acids including DNA (exoDNA). Thus, this study is to explore whether exoDNA in malignant pleural effusions (MPEs) could be a novel DNA source for mutation detection of epidermal growth factor receptor (EGFR).

**Methods:** In this study, 52 lung adenocarcinoma patients were enrolled, and EGFR mutation status was detected with tumor tissues as well as cell blocks and exosomes in MPEs. The sensitivity, specificity and consistency of EGFR detection using exosomes were evaluated, compared with gene detection using tumor tissues and cell blocks. And the clinical response of patients who were detected as EGFR mutation in exosomes and treated with EGFR tyrosine kinase inhibitor (EGFR-TKI) was explored.

**Results:** Gene detection using exosomes showed sensitivity of 100%, specificity of 96.55% and coincidence rate of 98.08% (Kappa = 0.961, *P* < 0.001), compared with detection using tumor tissues and cell blocks. After EGFR-TKI treatment, patients detected as EGFR mutation by exosomes showed efficacy rate of 83% and disease control rate of 100%. And patients who were detected as wild type in tumor tissues or cell blocks but EGFR mutation in exosomes turned up as PR or SD.

**Conclusions:** These results demonstrated that exoDNA in MPEs could be used as a DNA source for EGFR detection in lung adenocarcinoma.

## Introduction

Worldwide, lung cancer is the most common cancer among men in terms of both incidence and mortality, and nearly 50% are adenocarcinoma (1, 2). The mutation status of epidermal growth factor receptor (EGFR) acts as a significant molecular feature of lung adenocarcinoma patients and sensitizing EGFR mutations including in-frame deletion of exon 19 and L858R substitute mutation of exon 21 play a critical role in predicting the sensitivity to EGFR tyrosine kinase inhibitor (EGFR-TKI) (3–5). Therefore, EGFR mutation detection is extremely valuable for the diagnosis and treatment of lung cancer.

The DNA specimens for EGFR detection are particularly important, which are typically derived from tumor tissues, peripheral blood or cell blocks in malignant pleural effusions (MPEs). However, an invasive approach is required to obtain tumor tissues, which is painful, costly and time consuming. Moreover, detection by tumor tissues may not represent the complexity of tumor heterogeneity, both within a tumor and between a primary tumor and metastases (6). Accordingly, tumor tissues may not be the best source of tumor DNA. In recent years, cell free DNA (cfDNA) has shown broad prospects in molecule diagnosis, which can be easily purified from peripheral blood, with the size as large as 21 kb. And it has been reported that plasma cfDNA might be a valuable biomarker for tumor burden and prognosis in metastatic melanoma patients (7). Unfortunately, it has been verified in rats that large amounts of non-tumor DNA are released into blood during tumor progression particularly at early stages, which may lead to low specificity for tumor-related gene detection (8). As for cell blocks in MPEs, the low sensitivity of cytological examination and insufficiency of cell blocks become the main limiting factors to its practical application. For reason given above, it is urgent to seek a new source of tumor DNA for detection of EGFR mutation.

Exosomes are small vesicles (30–100 nm in diameter) secreted by various cell types and circulate in peripheral blood, urine, as well as pleural and peritoneal effusions (9, 10). Numerous proteins and nucleic acids that are representative of the secreting cells are present in exosomes, which are protected from degradation by the lipid bilayer (11, 12). Furthermore, there are excessive amount of exosomes released by tumor cells (13). The recent studies have disclosed the value of proteins and nucleic acids in tumor-derived exosomes as biomarkers for tumor diagnosis and prognosis (14–17). Although these studies predominantly focus on exosomal protein and microRNA, double-stranded genomic DNA (>10 kb) has been identified in exosomes (18–20). Moreover, it has been confirmed that DNA in exosomes (exoDNA) isolated from tumor cells is 20-fold more than that isolated from fibroblasts (18). And 80% of the exosomes purified from lung cancer patients contain EGFR, but the percentage is only about 2% for patients with chronic lung inflammation (21). All these characteristics make exosome become a valuable DNA source for gene detection in tumor diagnosis and treatment.

Therefore, in this study, EGFR mutation status was detected in tumor tissues, as well as cell blocks and exosomes in MPEs of lung adenocarcinoma patients, in order to explore whether exosome could be an ideal source of tumor DNA for EGFR mutation detection.

## Materials and Methods

### Patients and Samples

A prospective study was performed and 52 lung adenocarcinoma patients with MPEs were collected in the Fourth Medical Center of PLA General Hospital (Beijing, China) from January, 2015 to January, 2016. The inclusion criteria used for the enrolment of patients was: age between 18 and 90 years, confirmed diagnosis of lung adenocarcinoma with malignant pleural effusions by histological or cytological examination, ECOG score standard from 0 to 3, and no history of treatment with EGFR-TKI. The clinical information including age, gender and smoking habit was shown in Table 1. And the treatment and clinical response of all the patients were clarified in Supplementary Table 1.

**Table 1:** Clinical characteristics of patients enrolled in the study.

**Patient characteristics**	**Number of cases**
**AGE**	
<65	34
≥65	18
**GENDER**	
Male	22
Female	30
**SMOKING HABIT**	
Yes	18
No	34

MPEs were collected from all the patients. Exosomes in MPEs were extracted and enrolled in EGFR detection (*n* = 52). Cell blocks were also separated from MPEs and cytological examination was performed. The results revealed that the tumor cell detection rate was 63.46% (33/52), with 16 cases detected as no tumor cells and 3 cases as heterocysts. Therefore, 33 cases of cell blocks were enrolled in EGFR detection. Meanwhile, tumor tissues were obtained from 31 patients. Among them, 4 samples were derived from surgery and 27 samples were derived from biopsy of bronchus (*n* = 1), lung (*n* = 15), pleura (*n* = 2), subcutaneous metastatic nodule (*n* = 1), metastatic lymph node (*n* = 6), bone metastases (*n* = 1), and liver metastases (*n* = 1), all of which were enrolled in EGFR detection.

The follow-up data were collected by August, 2016, from 18 patients who were detected as EGFR mutation within exosomes and treated with EGFR-TKI of the first generation (Gefitinib or Icotinib). And the best treatment response was evaluated as complete remission (CR), partial remission (PR), stable disease (SD), or progressive disease (PD), according to the Response Evaluation Criteria in Solid Tumors (RECIST, version 1.1). The efficacy and disease control rate were defined as the percentage of CR + PR and CR + PR + SD, respectively. The evaluation was performed every month until disease progressed, patients died or the follow-up was completed.

This study was conducted in accordance with the World Medical Association's Declaration of Helsinki for experiments involving humans. All experimental protocols were approved by Ethics Committee of the Fourth Medical Center of PLA General Hospital and informed written consent was obtained from each patient prior to the recruitment.

### Exosome Extraction

Exosomes in MPEs were extracted using ExoQuick Exosome Precipitation Solution (System Biosciences, Palo Alto, CA, USA) according to the manufacturer's instructions. Pleural effusions were centrifuged at 3,000 g for 15 min to remove cellular debris and the supernatant was added with ExoQuick Exosome Precipitation Solution. After incubated overnight at 4°C, the ExoQuick/biofluid mixture was centrifuged at 1,500 g for 30 min. The supernatant was aspirated and residual mixture was centrifuged again at 1,500 g for 5 min. All traces of fluid were removed by aspiration without disturbing the precipitated exosomes in pellet. Finally, the exosome pellet resuspended in PBS was used immediately or stored at −80°C. All centrifugation was performed at 4°C.

### DNA Extraction and Evaluation

DNA was extracted from tumor tissues and cell blocks using MicroElute Genomic DNA kit (Omega Bio-tek, Norcross, GA, USA) as per the manufacturer's instructions. Samples were incubated with BL Buffer and OB Protease Solution at 55°C overnight and centrifuged at 13,000 g for 2 min. The supernatant was incubated with BL Buffer at 70°C for 10 min, and then added with 100% ethanol. The mixture was transferred into MicroElute LE DNA Column and centrifugation was performed at 13,000 g for 1 min. HBC Buffer and DNA Wash Buffer were added into the column in order and centrifugation was performed each time. After centrifugation at 13,000 g for 2 min, the empty column was added with Elution Buffer heated to 70°C. Centrifugation was performed at 13,000 g for 1 min, before which the column was placed at room temperature for 3 min. Finally, the collected DNA solution was used immediately or stored at −80°C.

In addition, exoDNA was extracted by QIAamp DNA Mini kit (QIAGEN, Hilden, Germany) after DNase I treatment, according to the manufacturer's instructions. Exosome pellet resuspended in PBS was incubated with DNase I and Buffer AL at 56°C for 10 min. After 100% ethanol was added, the mixture was transferred to the QIAamp Mini spin column. Centrifugation was performed at 6,000 g for 1 min and the filtrate was discarded. Buffer AW1 was added into the column and centrifugation was performed at 6,000 g for 1 min. Then Buffer AW2 was added into the column and centrifugation was performed at 20,000 g for 3 min. After centrifugation at 20,000 g for 1 min, the empty column was added with Buffer AE. Centrifugation was performed at 6,000 g for 1 min, before which the column was placed at room temperature for 1 min. Finally, the collected DNA solution was used immediately or stored at −80°C.

The concentration and purity of DNA derived from exosomes, cell blocks, or tumor tissues were assessed by Genova Nano micro-volume spectrophotometer (JENWAY, Staffordshire, UK). Besides, the size of double-stranded DNA fragments in exosomes was detected using Agilent 2100 bioanalyzer (Agilent Technologies, Palo Alto, GA, USA) and DNA 12000 reagent kit (Agilent Technologies).

### Analysis of EGFR Mutation

Human EGFR Gene Mutation Detection kit (YZYBIO, Wuhan, Hubei province, China) was used to define EGFR mutation status through amplification refractory mutation system according to the manufacturer's instructions. This kit could detect 11 types of EGFR mutation, including G719X (G719S, G719C, or G719A) substitute mutation in exon 18, deletion mutation in exon 19, L858R, and L861Q substitute mutations in exon 21, S768I, and T790M substitute mutations in exon 20, as well as 3 types of insertion mutations in exon 20. Therefore, in this study, “wild type” meant EGFR without mutations as mentioned above. Meanwhile, a house-keeping gene was used as the quality control. And name of the house-keeping gene, as well as sequences of the primers and probes, were confidential for users. According to the manufacturer's information, the kit can detect EGFR mutations that account for 1% of 20 ng genomic DNA, using paraffin tissue sections from lung cancer patients.

Quantitative PCR was performed on ABI Prism 7900HT Detection System (Thermo Fisher Scientific, Waltham, MA, USA). The PCR cycling conditions were as follows: (i) 37°C for 10 min; (ii) 95°C for 5 min; (iii) 40 cycles of 95°C for 15 s, 60°C for 1 min. Ct value was determined by the amplification plot and ΔCt was defined as the difference between the Ct value of specific mutation type and the quality control. Finally, it was considered positive for EGFR mutation when ΔCt was less than specific value depending on different mutation types (9 for G719X, 8 for S768I and T790M, 7 for other mutations). And the method to determine the threshold was confidential for users.

### Statistical Analysis

Consistency test was performed to evaluate whether EGFR mutation detection within exosomes was in accordance with gene detection using tumor tissues and cell blocks, and Kappa > 0.75 was indicative of high consistency. A value of *P* < 0.05 was considered statistically significant. Data analysis was performed using SPSS software (version 22.0; SPSS Inc., Chicago, IL, USA; RID: SCR_002865).

## Results

### Features of ExoDNA

**Figure 1:**
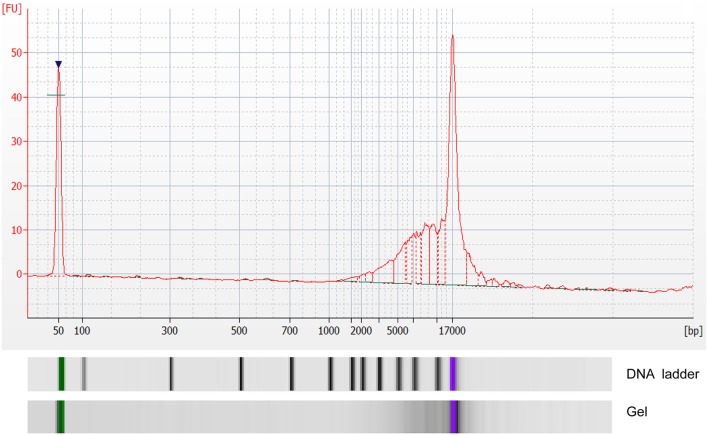
Analysis of double-stranded DNA fragments in exosomes by Agilent 2100 bioanalyzer. X-axis indicated number of base pairs (bp) and Y-axis indicated fluorescence unit (FU).

The concentration of exoDNA derived from MPEs ranged from 1.74 to 20.96 ng/μl [M (P25, P75) = 5.64 (2.96, 7.98) ng/μl]. Moreover, exoDNA was enriched in double-stranded DNA fragments at the size of 17 kb (Figure 1).

### EGFR Mutation Status Detected in Different Sample Types

**Figure 2:**
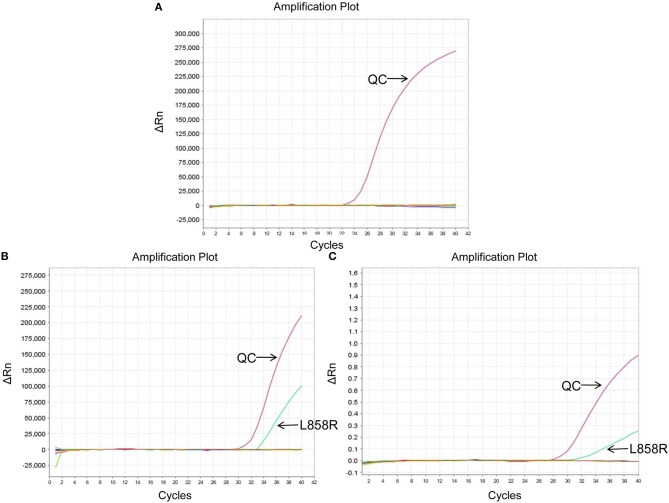
EGFR mutation status of patient No.23 using different sample types. **(A)** Wild type in tumor tissues. **(B)** Exon 21 L858R in cell blocks. **(C)** Exon 21 L858R in exosomes. ΔCt was defined as the difference between the Ct value of specific mutation type and QC. And it was considered positive for exon 21 L858R when ΔCt was <7. QC, quality control (a house-keeping gene).

The EGFR mutation status detected in different sample types were shown in Supplementary Table 1. The EGFR mutation rate in tumor tissues was 32.26% (10/31), including 7 cases of deletion mutation in exon 19 (exon 19 Del) and 3 cases of L858R substitute mutation in exon 21 (exon 21 L858R). As for cell blocks in MPEs, the EGFR mutation rate was 51.52% (17/33, including 9 cases of exon 19 Del and 8 cases of exon 21 L858R). Compared with matched tumor tissues (*n* = 12), the diagnostic performance of cell blocks showed sensitivity of 100% (3/3) and specificity of 88.89% (8/9). And the concordance rate was 91.67% (11/12, Kappa = 0.8, *P* = 0.005; Table 2), indicating high consistency. In addition, patient No.23 was detected as exon 21 L858R in cell blocks but wild type in tumor tissues (Supplementary Table 1; Figures 2A,B).

**Table 2:** EGFR mutation detection by tumor tissues and matched cell blocks in MPEs.

**EGFR mutation status**		**Tissues**		**Total**	**Kappa coefficient**
		**M**	**WT**		
Cell blocks	M	3	1	4	0.8 (*P* < 0.001^*^)
	WT	0	8	8	
Total		3	9	12	

EGFR, epidermal growth factor receptor; MPEs, malignant pleural effusions; M, mutant; WT, wild type.

* *P value was calculated by Consistency test*.

**Figure 3:**
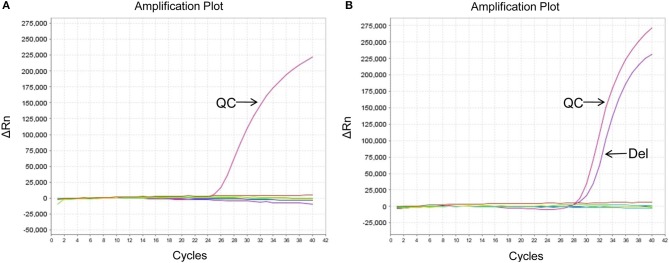
EGFR mutation status of patient No.17 using different sample types. **(A)** Wild type in cell blocks. **(B)** Exon 19 Del in exosomes. ΔCt was defined as the difference between the Ct value of specific mutation type and QC. And it was considered positive for exon 19 Del when ΔCt was <7. QC, quality control (a house-keeping gene).

The EGFR mutation status was also detected in exosomes derived from MPEs, and the results indicated that the mutation rate was 46.15% (24/52, including 14 cases of exon 19 Del and 10 cases of exon 21 L858R). When compared with matched tumor tissues (*n* = 31), EGFR detection using exosomes showed sensitivity of 100% (10/10), specificity of 95.24% (20/21) and concordance rate of 96.77% (30/31, Kappa = 0.928, *P* < 0.001; Table 3). And patient No.23 was detected as exon 21 L858R in exosomes but wild type in tumor tissues (Supplementary Table 1; Figures 2A,C). When compared with matched cell blocks (*n* = 33), gene detection using exosomes showed sensitivity of 100% (16/16), specificity of 94.12% (16/17) and concordance rate of 96.97% (32/33, Kappa = 0.939, *P* < 0.001; Table 4). And patient No.17 was detected as exon 19 Del in exosomes but wild type in cell blocks (Supplementary Table 1; Figure 3). Moreover, when compared with the combination of tumor tissues and cell blocks (*n* = 52), the diagnostic performance of exosomes showed sensitivity of 100% (23/23) and specificity of 96.55% (28/29). And the concordance rate was 98.08% (51/52, Kappa = 0.961, *P* < 0.001; Table 5), indicating high consistency.

**Table 3:** EGFR mutation detection by exosomes in MPEs and matched tumor tissues.

**EGFR mutation status**		**Tissues**		**Total**	**Kappa coefficient**
		**M**	**WT**		
Exosomes	M	10	1	11	0.928 (*P* < 0.001^*^)
	WT	0	20	20	
Total		10	21	31	

EGFR, epidermal growth factor receptor; MPEs, malignant pleural effusions; M, mutant; WT, wild type.

* *P value was calculated by Consistency test*.

**Table 4:** EGFR mutation detection by exosomes and cell blocks in MPEs.

**EGFR mutation status**		**Cell blocks**		**Total**	**Kappa coefficient**
		**M**	**WT**		
Exosomes	M	16	1	17	0.939 (*P* < 0.001^*^)
	WT	0	16	16	
Total		16	17	33	

EGFR, epidermal growth factor receptor; MPEs, malignant pleural effusions; M, mutant; WT, wild type.

* *P value was calculated by Consistency test*.

**Table 5:** EGFR mutation detection by tumor tissues, as well as cell blocks and exosomes in MPEs.

**EGFR mutation status**		**Tissues and cell blocks**		**Total**	**Kappa coefficient**
		**M**	**WT**		
Exosomes	M	23^#^	1	24	0.961 (*P* < 0.001^*^)
	WT	0	28	28	
Total		23	29	52	

EGFR, epidermal growth factor receptor; MPEs, malignant pleural effusions; M, mutant; WT, wild type;

# Among the 23 cases, patient No.23 was detected as exon 21 L858R in cell blocks but wild type in tumor tissues.

* *P value was calculated by Consistency test*.

### Clinical Response of Patients Who Were Detected As EGFR Mutation Within Exosomes and Treated With EGFR-TKI

The follow-up data were collected from 18 patients who were detected as EGFR mutation within exosomes and treated with EGFR-TKI. As shown in Supplementary Table 1, there were 15 cases of PR and 3 cases of SD. Interestingly, patient No.23 was detected as exon 21 L858R in exosomes and cell blocks but wild type in tumor tissues, and was treated with Gefitinib as 2nd line treatment. Computed axial tomography was performed to evaluate the clinical response which turned up as PR. And the progression-free survival time was 9 months. Meanwhile, patient No.17 was detected as exon 19 Del in exosomes but wild type in cell blocks, and was treated with Icotinib as 3rd line treatment. The clinical response turned up as SD, with the progression-free survival time being 4 months (Supplementary Table 1). These results demonstrated that EGFR mutation detection in exosomes may have important practical value in predicting the therapeutic effect of EGFR-TKI.

## Discussion

In this study, EGFR mutation status was detected in tumor tissues as well as cell blocks and exosomes in MPEs, and the diagnostic value of exosome as a new DNA source for EGFR mutation detection was explored. The results indicated great consistency among different sample types and perfect diagnostic performance of exosomes in MPEs.

It has been demonstrated that the EGFR mutation rate of lung adenocarcinoma patients in Asia is ~51.4%, mainly detected in female and non-smokers. And patients with exon 19 Del and exon 21 L858R accounted for 24.6 and 22.8%, respectively (22). In this study, the EGFR mutation rate detected in tumor tissues, cell blocks, and exosomes were 32.26, 51.52, and 46.15%, respectively, with the mutation types focusing on exon 19 Del and exon 21 L858R, which was in accordance with previous study.

In our study, features of exoDNA derived from MPEs were examined and double-stranded DNA fragments at the size of 17 kb were detected. This result was consistent with conclusion from other researchers that double-stranded genomic DNA (>10 kb) has been identified in exosomes, providing the possibility that exosome may be a novel DNA source for gene detection (18–20). Moreover, gene detection in exosomes showed great consistency, no matter compared with tumor tissues, cell blocks or both of them, indicating perfect performance of exosome as a tumor DNA source for EGFR detection.

Detection of EGFR mutation status has been proved to play an important role in predicting the treatment response to EGFR-TKI, especially when sensitizing mutations such as exon 19 Del and exon 21 L858R are concerned. It has been demonstrated that non-small cell lung cancer patients with EGFR mutation had higher objective response rate and longer median progression-free survival time than those with wild type EGFR, no matter detected in tumor tissues or plasma DNA (23). And Jeng-Sen Tseng et al. also indicated that EGFR mutation status in plasma cfDNA could serve as a predictor of EGFR-TKI efficacy in patients with lung adenocarcinoma (24). The DNA specimens are of vital importance for EGFR detection and it has been reported that the combination of exosomal RNA/DNA and cfDNA in plasma for T790M detection has higher sensitivity and specificity compared with historical cohorts using cfDNA alone (25). In this study, patients who were detected as EGFR mutation within exosomes showed high efficacy and disease control rate after EGFR-TKI treatment. Moreover, patients who were detected as EGFR mutation by exosomes but wild type by cell blocks or tumor tissues finally indicated as PR or SD. These results demonstrated that EGFR mutation detection in exosomes may have important practical value in tumor diagnosis and treatment.

At last, there were still some limitations in this study. On one hand, although exosomes have several advantages as a new tumor DNA source, the extraction of exosomes may still limit their clinical application. Ultracentrifugation is the traditional method to isolate exosomes, which is time consuming and needs special equipment. Moreover, repeated ultracentrifugation steps can reduce the quality of exosome preparations leading to lower yield (26). New approaches have been developed and several commercial kits are available, which are user-friendly and do not need special equipment, but are usually expensive. Therefore, it is urgent to seek a proper method of exosome extraction which is cheap and convenient to perform in clinical detection. On the other, ARMS-qPCR was used for EGFR mutation detection, which is one of the most popular approaches used in clinical detection. Compared with next-generation sequencing (NGS), ARMS-qPCR is easy to perform and its cost is relatively low. However, it is not suitable for unknown mutations and the sensitivity of ARMS-qPCR is lower than that of NGS, which may limit its application in the future.In summary, the feasibility of exosome in MPEs as a novel source of tumor DNA was investigated in this study. EGFR mutation detection in exosomes showed great consistency when compared with gene detection in tumor tissues and cell blocks, and may have important practical value in predicting the treatment response to EGFR-TKI. As a non-invasive approach, gene detection in exosomes would have wide application prospect in diagnosis and treatment of tumors.

## Data Availability Statement

The raw data supporting the conclusions of this manuscript will be made available by the authors, without undue reservation, to any qualified researcher.

## Ethics Statement

The studies involving human participants were reviewed and approved by Ethics Committee of the Fourth Medical Center of PLA General Hospital. The patients/participants provided their written informed consent to participate in this study.

## Author Contributions

WX and JZ contributed to design of the study and gave final approval of the manuscript to be submitted. XQ, QL, and SZ contributed to acquisition of the data. FW, FZ, and HZhan contributed to analysis and interpretation of the data. RW, QiW, and YH performed the statistical analysis. JY, HZhao, and QiaW drafted and revised the manuscript. XZ, XP, and GL contributed to the collection and management of samples and patients' information.

### Conflict of Interest

The authors declare that the research was conducted in the absence of any commercial or financial relationships that could be construed as a potential conflict of interest.

## Abbreviations

EGFR: epidermal growth factor receptor
EGFR-TKI: EGFR tyrosine kinase inhibitor
MPEs: malignant pleural effusions
cfDNA: cell free DNA
exoDNA: exosomal DNA
CR: complete remission
PR: partial remission
SD: stable disease
PD: progressive disease
exon 19 Del: deletion mutation in exon 19
exon 21 L858R: L858R substitute mutation in exon 21.
