# Supplementary material for: Double-Stranded DNA in Exosomes of Malignant Pleural Effusions as a Novel DNA Source for EGFR Mutation Detection in Lung Adenocarcinoma
Source: Front Oncol. 2019 Sep 25;9:931. doi: 10.3389/fonc.2019.00931 (PMC6773809; doi:10.3389/fonc.2019.00931)
Supplement: Supplementary file 1 [file Table_1.docx]

Supplementary Material

**Supplementary Table 1.** EGFR mutation status detected in different sample types and EGFR-TKI treatment response of patients.

| **No.** | **Age** | **Sex** | **Smoking habit** | **Tumor tissues** | **Cell blocks** | **Exosomes** | **EGFR-TKI** | **PFS** |
| --- | --- | --- | --- | --- | --- | --- | --- | --- |
| 1 | 74 | male | Yes | × | WT | WT | unused | × |
| 2 | 61 | female | No | L858R | L858R | L858R | unused | × |
| 3 | 49 | female | No | × | L858R | L858R | 2nd line, PR | 13 |
| 4 | 75 | female | No | × | L858R | L858R | 1st line, PR | 17 |
| 5 | 43 | male | No | DEL 19 | × | DEL 19 | 2nd line, PR | 7 |
| 6 | 63 | female | No | WT | WT | WT | unused | × |
| 7 | 47 | female | No | DEL 19 | × | DEL 19 | 2nd line, PR | 13 |
| 8 | 74 | female | No | × | L858R | L858R | 1st line, PR | 16 |
| 9 | 35 | male | No | WT | × | WT | unused | × |
| 10 | 47 | male | Yes | × | DEL 19 | DEL 19 | 1st line, SD | 3 |
| 11 | 73 | female | No | DEL 19 | DEL 19 | DEL 19 | 1st line, PR | 13 |
| 12 | 65 | female | No | × | DEL 19 | DEL 19 | 1st line, PR | 5 |
| 13 | 57 | female | No | WT | × | WT | 2nd line, SD | 2 |
| 14 | 54 | female | No | WT | × | WT | 2nd line, SD | 7 |
| 15 | 50 | female | No | × | WT | WT | 2nd line, PR | 9 |
| 16 | 45 | female | No | WT | × | WT | 1st line, PD | 1 |
| 17 | 30 | female | No | × | WT | DEL 19 | 3rd line, SD | 4 |
| 18 | 60 | male | Yes | × | L858R | L858R | unused | × |
| 19 | 73 | female | Yes | WT | × | WT | 1st line, PD | 1 |
| 20 | 60 | female | No | WT | WT | WT | unused | × |
| 21 | 80 | male | No | WT | × | WT | unused | × |
| 22 | 55 | female | No | WT | WT | WT | unused | × |
| 23 | 52 | male | Yes | WT | L858R | L858R | 2nd line, PR | 9 |
| 24 | 51 | male | Yes | L858R | × | L858R | 1st line, PR | 7 |
| 25 | 63 | male | Yes | WT | WT | WT | 2nd line, PR | 4 |
| 26 | 57 | male | Yes | DEL 19 | × | DEL 19 | unused | × |
| 27 | 71 | female | No | × | L858R | L858R | unused | × |
| 28 | 57 | male | Yes | WT | WT | WT | 1st line, PD | 0.5 |
| 29 | 84 | male | No | × | L858R | L858R | 1st line, PR | 12 |
| 30 | 50 | male | Yes | WT | WT | WT | 3rd line, PD | 0.7 |
| 31 | 54 | male | Yes | × | DEL 19 | DEL 19 | unused | × |
| 32 | 71 | male | Yes | WT | × | WT | unused | × |
| 33 | 67 | male | No | DEL 19 | × | DEL 19 | 1st line, PR | 15 |
| 34 | 54 | female | No | × | DEL 19 | DEL 19 | 3rd line, PR | 2 |
| 35 | 74 | female | No | × | WT | WT | 2nd line, PD | 1 |
| 36 | 52 | female | Yes | DEL 19 | × | DEL 19 | unused | × |
| 37 | 59 | male | No | × | DEL 19 | DEL 19 | 1st line, SD | 12 |
| 38 | 62 | female | No | WT | × | WT | unused | × |
| 39 | 68 | male | Yes | × | WT | WT | unused | × |
| 40 | 56 | female | No | WT | × | WT | unused | × |
| 41 | 59 | female | No | × | WT | WT | 1st line, PD | 1 |
| 42 | 39 | female | No | WT | WT | WT | unused | × |
| 43 | 60 | female | No | WT | × | WT | unused | × |
| 44 | 60 | female | No | L858R | × | L858R | 2nd line, PR | 8 |
| 45 | 60 | male | Yes | WT | WT | WT | unused | × |
| 46 | 63 | male | No | × | WT | WT | unused | × |
| 47 | 45 | female | No | DEL 19 | DEL 19 | DEL 19 | 2nd line, PR | 4 |
| 48 | 68 | female | No | × | WT | WT | unused | × |
| 49 | 67 | male | Yes | WT | × | WT | unused | × |
| 50 | 72 | female | Yes | × | WT | WT | unused | × |
| 51 | 74 | male | Yes | WT | × | WT | unused | × |
| 52 | 69 | female | No | × | DEL 19 | DEL 19 | 2nd line, PR | 5 |

*EGFR, epidermal growth factor receptor; TKI, tyrosine kinase inhibitor; WT, wild type; ×, No detection or no collection; PR, partial remission; SD, stable disease; PD, progressive disease; DEL 19, deletion mutation in exon 19; L858R, L858R substitute mutation in exon 21; PFS, progression-free survival time (months).*
